# hCLP46 increases Smad3 protein stability via inhibiting its ubiquitin-proteasomal degradation

**DOI:** 10.1007/s13238-015-0174-0

**Published:** 2015-06-10

**Authors:** Yingying Xing, Qiaoyun Chu, Run Feng, Wei Wang, Lixin Liu, Zhongbing Lu

**Affiliations:** College of Life Sciences, University of Chinese Academy of Science, Beijing, 100049 China; Key Laboratory for Polymeric Composite and Functional Materials of Ministry of Education, School of Chemistry and Chemical Engineering, Sun Yat-Sen University, Guangzhou, 510275 China; School of Medical Science, Edith Cowan University, Joondalup, WA 6027 Australia; Department of Biochemistry and Molecular Biology, Capital Medical University, Beijing, 10069 China

**Dear Editor,**

hCLP46 (human CAP10-like protein 46 kDa) was initially isolated and identified from human acute myeloid leukemia transformed from myelodysplastic syndrome (MDS-AML) CD34^+^ cells (Teng et al., 2006) and we demonstrated previously that hCLP46 is abnormally expressed in many hematopoietic malignancies (Wang et al., 2010). Studies from its *Drosophila* homolog, Rumi, suggested that Notch is a potential target of hCLP46 (Acar et al., 2008). We also found that overexpression of hCLP46 enhances Notch activation and regulates cell proliferation in a cell type-dependent manner (Ma et al., 2011; Chu et al., 2013). However, hCLP46^−/−^ embryos show more severe phenotypes compared to those displayed by other global regulators of canonical Notch signaling, suggesting that hCLP46 is likely to have additional important targets during mammalian development (Fernandez-Valdivia et al., 2011). Based on the crosstalk between Notch and the transforming growth factor-β (TGF-β) signaling, we proposed that hCLP46 might be involved in TGF-β signal regulation, but the detail mechanism remains unclear.

With the full length or truncated plasmids encoding hCLP46 1–120 aa (no CAP10 domain) or hCLP46 121–392 aa (with CAP10 domain), we found that overexpression of hCLP46 1–120 aa had no obvious effect on Smad3 expression, whereas both hCLP46 121–392 aa and hCLP46-full length significantly increased Smad3 protein expression, suggesting that hCLP46 increases Smad3 expression in a CAP10 domain dependent manner (Fig. 1A and 1B). To determine the mechanism through which hCLP46 regulates Smad3 expression, we generated a stable cell line inducibly overexpressing hCLP46, which is named as 293TRex-hCLP46 hereinafter. When cells were incubated with 0.5 μg/mL Tetracycline (Tet) for 24 h, the pronounced induction of hCLP46 resulted in 85% increase of Smad3 expression (Fig. 1C and 1D). We then examined Smad3 protein turnover in 293TRex-hCLP46 cells by blocking protein synthesis. In Tet off cells, administration of CHX (50 μg/mL) caused a remarkable decrease of Smad3 in a time dependent manner (Fig. 1C and 1D). The half-life is only about 0.5 h, suggesting that endogenous Smad3 undergoes fast degradation at the steady state. However, the half-life of Smad3 protein was significantly longer in Tet on cells as compared with that observed in Tet off cells, suggesting that hCLP46 could increase Smad3 protein stability (Fig. 1C and 1D). When endogenous hCLP46 was knocked down by siRNA, significantly reduction in Smad3 expression was observed (Fig. 1E). It is notable that the mRNA levels of Smad3 were not affected by overexpression or knockdown of hCLP46 (Fig. S1A and S1B). To determine whether the degradation of Smad3 is mediated by proteasome, we treated 293TRex-hCLP46 cells with 20 μmol/L 26S proteasome inhibitor MG-132 for 3 h, which resulted in a two-fold increase in Smad3 expression in Tet off cells (Fig. 1F and 1G). In the presence of MG132, hCLP46 overexpression still significantly increased Smad3 protein level whereas hCLP46 knockdown had no obvious effect on Smad3 expression (Fig. 1F and 1G). Consistently, we found that Smad3 was polyubiquitinated in cells treated with MG132 and the ubiquitination of Smad3 was attenuated by overexpression of hCLP46 (Fig. 1H), but enhanced by knockdown of endogenous hCLP46 (Fig. 1I), suggesting that hCLP46 increases Smad3 expression and protein stability through inhibiting proteasomal degradation of Smad3.

**Figure 1:**
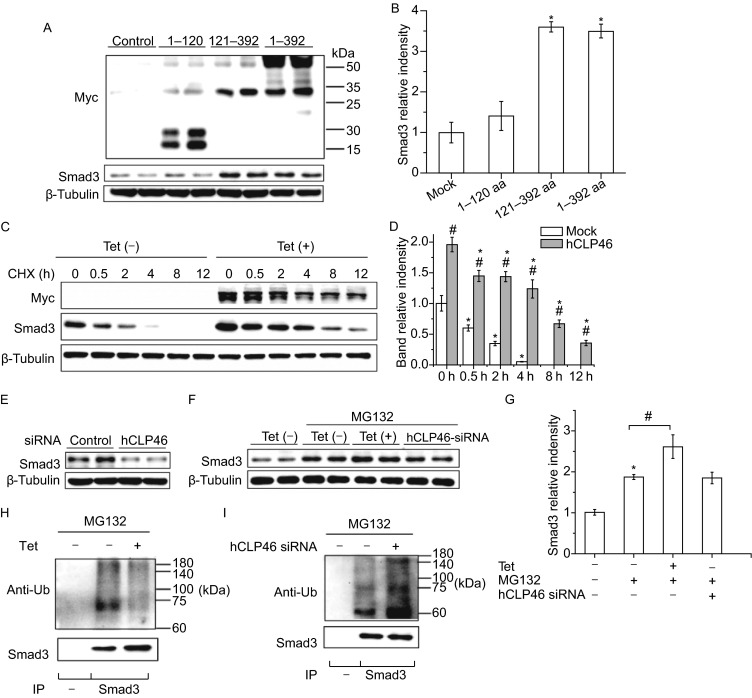
**hCLP46 increases Smad3 expression by preventing its proteasomal degradation in 293TRex cells**. (A) 293TRex cells were transfected with full length of hCLP46 or truncated plasmids encoding hCLP46 1–120 aa or hCLP46 121–392 aa for 48 h, the protein levels of Myc and Smad3 were determined by Western blot. (C) 293TRex-hCLP46 cells were cultured in absence or presence of 0.5 μg/mL Tet for 24 h and 50 μg/mL CHX was then added as indicated. Total cell lysates were probed for Myc and Smad3. (E) After transfected with control or hCLP46 specific siRNA for 72 h, the protein level of Smad3 was determined. (F) 293TRex-hCLP46 cells were incubated with or without 0.5 μg/mL Tet for 24 h or transfected with hCLP46 specific siRNA for 72 h, 20 μmol/L MG132 was then added for 3 h. Cell lysates were examined by Western blot for Smad3 or used for immunoprecipitation (IP) with no antibody (−) or anti-Smad3 then probed with ubiquitin antibody (anti-Ub) (H and I). β-Tubulin was used as a loading control in (A), (C), (E) and (F). Immunoblot band intensities were quantified using loading controls (B, D and G). *n* = 4 in each group. *Indicates *P* < 0.05 comparing to control and hCLP46 plasmids transfected or CHX/MG132 treated cells. ^#^Indicates *P* < 0.05 comparing to control and hCLP46 overexpressed cells

We then attempted to evaluate the impact of hCLP46 on TGF-β signaling. Overexpression of hCLP46 inhibited cell viability at basal condition and further exacerbated TGF-β1 induced cell viability inhibition (2 ng/mL, 24 h) (Fig. 2A). In contrast, knockdown of hCLP46 by siRNA increased cell viability and almost totally blocked the inhibition of cell proliferation by TGF-β1 (Fig. 2B). In addition, TGF-β1 treatment significantly increased the expression of two cell cycle inhibitors (p21 and p27) as compared to control cells, which were further enhanced by overexpression of hCLP46 (Fig. 2C and 2D), whereas attenuated by knockdown of hCLP46 (Fig. 2E and 2F), suggesting that hCLP46 enhances TGF-β signaling by modulating Smad3 expression.

**Figure 2:**
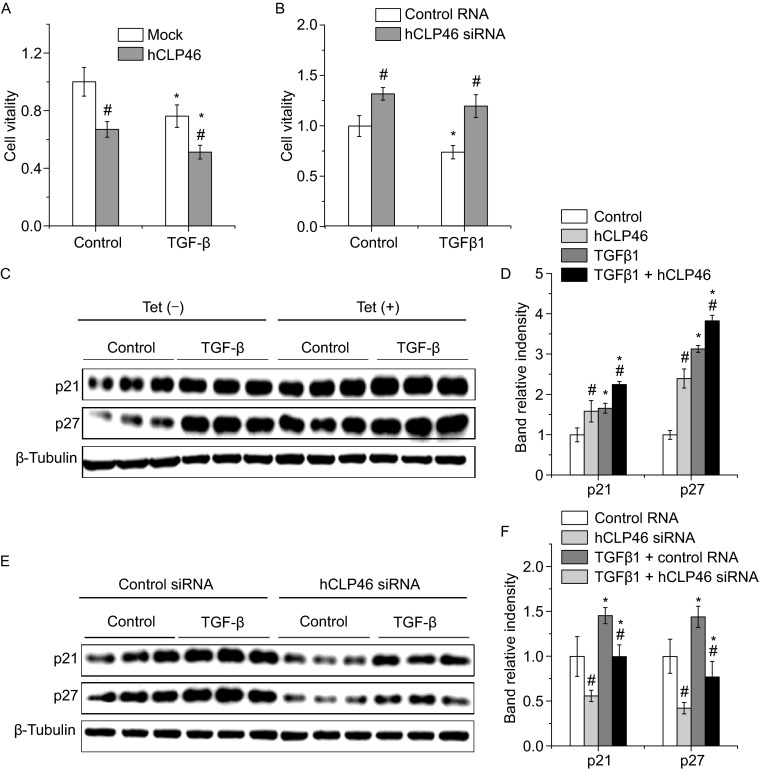
**hCLP46 enhanced TGF-β1 induced cell growth arrest and up-regulation of cell cycle inhibitors**. (A) 293TRex-hCLP46 cells were cultured in absence or presence of 0.5 μg/mL Tet for 24 h and 2 ng/mL TGF-β1 was then added for 24 h. Cell viability was determined by MTT method. (B) 293TRex-hCLP46 cells were maintained in absence of Tet and transfected with control or hCLP46 specific siRNA for 48 h. Then cells were treated with 2 ng/mL TGF-β1 for additional 24 h. Cell viability was determined by MTT method. In (A) and (B), data were collected from 8 independent experiments. (C) Lysates from control, Tet, TGF-β1 and Tet plus TGF-β1 treated cells were probed for p21 and p27. (E) The protein levels of p21 and p27 were also determined in lysates from control, hCLP46 siRNA, TGF-β1 and hCLP46 siRNA plus TGF-β1 treated cells. β-Tubulin was used as a loading control in (C) and (E). Immunoblot band intensities were quantified using loading controls (D and F). *n* = 3 in each group. *Indicates *P* < 0.05 comparing to control and TGF-β1 treated cells. ^#^Indicates *P* < 0.05 comparing to control and hCLP46 overexpressed or hCLP46 siRNA transfected cells

As Notch signaling might be involved in Smad3 regulation, we treated cells with DAPT (2 μmol/L, 12 h) or EDTA (5 mmol/L, 15 min and then replaced with fresh DMEM plus 10% FBS and cultured for additional 6 h) to inhibit or activate Notch signaling respectively. DAPT treatment resulted in dramatically decrease of the NICD expression, but has no effect on Smad3 expression (Fig. S2A–C). EDTA significantly increased NICD and Smad3 levels. However, cells with hCLP46 overexpression still had more Smad3 expression than that of control cells (Fig. S2D–F). Together, these data suggest that hCLP46 regulates Smad3 expression is not affected by Notch signaling.

As the primary intracellular transducer, Smad3 is a critical mediator of the cytostatic response to TGF-β (Zhang et al., 2014). Evidence for this comes from the observation that a variety of primary cells from Smad3-null mice are partially resistant to TGF-β induced growth arrest (Datto et al., 1999; Yang et al., 1999) and exogenous overexpression of Smad3 sensitizes cells to TGF-β induced growth arrest and apoptosis (Wildey et al., 2003). Importantly, it was found that treatment with the proteasome inhibitor caused an accumulation of Smad3 protein in absence of TGF-β1, suggesting that not only in response to TGF-β but also in a steady state, the level of Smad3 is regulated by the proteasome pathway (Inoue et al., 2004). In addition, several studies demonstrated that the steady-state stability of Smad3 is an important determinant of cellular sensitivity to TGF-β (Guo et al., 2008) and the U-box-containing carboxyl terminus of Hsc70-interacting protein has been identified as an E3 ubiquitin ligase to degrade Smad3 at steady state (Xin et al., 2005). In agreement with those findings, we demonstrated that hCLP46 modulates Smad3 protein stability by inhibiting its proteasomal degradation and consequently enhances cellular sensitivity to the TGF-β signal. Our findings suggest a new function of hCLP46 in modulating critical TGF-β/Smad3-regulated processes during development and tumor progression.

## Electronic supplementary material

Supplementary material 1 (PDF 561 kb)
