# Erratum to: hCLP46 increases Smad3 protein stability via inhibiting its ubiquitin-proteasomal degradation

**DOI:** 10.1007/s13238-015-0186-9

**Published:** 2015-07-07

**Authors:** Yingying Xing, Qiaoyun Chu, Run Feng, Wei Wang, Lixin Liu, Zhongbing Lu

**Affiliations:** 1 College of Life Sciences, University of Chinese Academy of Science, Beijing, 100049 China; 2 Key Laboratory for Polymeric Composite and Functional Materials of Ministry of Education, School of Chemistry and Chemical Engineering, Sun Yat-Sen University, Guangzhou, 510275 China; 3 Department of Biochemistry and Molecular Biology, Capital Medical University, Beijing, 100069 China; 4 School of Medical Science, Edith Cowan University, Joondalup, WA 6027 Australia

## Erratum to: Protein Cell DOI 10.1007/s13238-015-0174-0

In the original publication of the article, co-authors affiliations are appeared incorrectly. Correct affiliations are provided in this erratum.

