# Supplementary material for: hCLP46 increases Smad3 protein stability via inhibiting its ubiquitin-proteasomal degradation
Source: Protein Cell. 2015 Jun 10;6(10):767–70. doi: 10.1007/s13238-015-0174-0 (PMC4598319; doi:10.1007/s13238-015-0174-0)
Supplement: Supplementary file 1 — Supplementary material 1 (PDF 561 kb) [file 13238_2015_174_MOESM1_ESM.pdf]

SUPPLEMENTARY MATERIALS

hCLP46 enhances TGF-β signaling by increasing Smad3 protein stability

Yingying Xing[1#], Qiaoyun Chu[1, 2#], Run Feng[1], Wei Wang[3], Lixin Liu[1, 4*], Zhongbing Lu[1*]

[1] College of Life Sciences, University of Chinese Academy of Science, Beijing, 100049, China

[2] Department of Biochemistry and Molecular Biology, Capital Medical University, Beijing, 10069, China

[3] School of Medical Science, Edith Cowan University, Joondalup, WA, 6027, Australia

[4] Key Laboratory for Polymeric Composite and Functional Materials of Ministry of Education, School of Chemistry and Chemical Engineering, Sun Yat-Sen University, Guangzhou, 510275, China

# These authors contributed equally to this work

* Corresponding author:

Lixin Liu, PhD

School of Chemistry and Chemical Engineering, Sun Yet-sen University

NO. 135, Xingang Xi Road, Guangzhou, 510275, China

Tel. +86 15622237496; Fax +86 2084113261

Email: liulixin@mail.sysu.edu.cn

Or

Zhongbing Lu, Ph.D

College of Life Science, University of Chinese Academy of Science

19A Yuquanlu, Beijing, 100049, China

Fax: 86(10)88256079; Tel: 86(10)88256713

E-mail: luzhongbing@ucas.ac.cn

Material and methods

The plasmid encoding hCLP46 open reading frame (ORF), hCLP46-specific siRNA and control siRNA were produced as previously described (Ma et al., 2011). The DNA sequence encoding the 1-120aa and 121-392aa of hCLP46 were amplified by PCR and inserted into the pcDNA4/TO/Myc vector (Invitrogen, Carlsbad, CA, USA) using BamHI and XhoI sites incorporated into primers (underlined). *For 1-120, forward primer: 5'-AAA GGATCC GCCACCATGGAGTGGTGGGCTAGC-3, reverse primer: 5'-AGA CTCGAG CGCCCGATCACTTCCAAAAT-3. For 121-392, forward primer: 5'-AAA GGATCC GCCACC ATG CGTCTCCCTGACATG-3, 5-5'-AGA CTCGAG CTACAGATCCTCTTCTGAGAT-3.* DAPT (N-[N-(3,5-difluorophenacetyl)-1-alanyl]-S-phenylglycine t-butyl ester) and MG132 were purchased from Sigma Chemical Co. (St. Louis, MO, USA). Cycloheximide (CHX) and TGF-β1 were purchased from Cayman Chemical Co. (Ann Arbor, MI, USA) and PeproTech Inc. (Rocky Hill, NJ, USA) respectively. Antibodies against the following proteins were used: Smad3, ubiquitin, p21, p27, β-tubulin and Myc-tag (Cell Signaling Technology, Danvers, MA, USA).

Cell culture, transfections and growth assay

Cells were cultured in Dulbecco's modified Eagle's medium (DMEM; Gibco BRL, Gaithersburg, MD, USA) supplemented with 10% FBS and maintained at 37 ℃ under humidified conditions and 5% CO_2. Plasmids and siRNA were transfected using Lipofectamine 2000 (Invitrogen) according to the manufacturer's instructions. MTT method was used for cell growth assay as previous reported(Chu et al., 2013).

Gene expression analysis with quantitative real-time PCR

Total RNA was extracted from cells using Trizol reagent, and cDNA was synthesized with SuperScript III First-Strand Synthesis Super-Mix for quantitative PCR (Invitrogen). The primers used are as follows: hCLP46: forward 5’-GATATCATGTATCCTGCTTG-3’; reverse 5’-TTTTCCATGGCCACTGTGCTG-3’. Smad3: forward 5’-AAACCAGGCTGGCTAAACAAGTG-3’; reverse 5’-GCAACAGCAGTGAAGGTG-3’. β-actin: forward 5’-GTGACGTGGACATCCGCAAA GAC-3’ ; reverse: 5’-TCAAGAAAGGGTGTAACGCAACTAA-3’. Quantitative PCR was carried out in a Rotor-Gene 6000 centrifugal real-time cycler (Corbett Research, CA) using the kit of Platinum SYBR Green qPCR Super-Mix-UDG with ROX. The cycling conditions were as follows: initial denaturation at 95 ℃ for 2 min, followed by 45 cycles of 15 s at 95 ℃, 30 s at 58 ℃, and 30 s at 72 ℃.

Western blotting

The cells were washed with ice-cold PBS and then lysed by buffer (50 mM Tris-Cl, 150mM NaCl, 100 μg/ml phenylmethylsulfonyl fluoride, protease and phosphatase inhibitor cocktail from Roche and 1%Triton X-100) on ice for 30 min. After centrifugation at 12,000 g for 20min at 4 ℃, the supernatant was used for western blot analysis as previous described.

Statistical analysis

All values are expressed as mean ± standard error. Differences between control and target data sets were determined using independent sample t-tests, with P-values less than or equal to 0.05 being considered statistically significant.

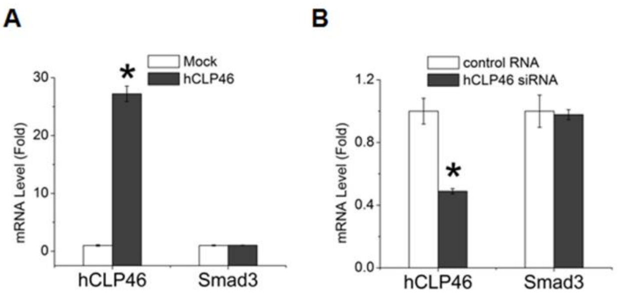

Figure S1. The mRNA levels of Smad3 were not affected by hCLP46. (A) After cultured for 24h, the mRNA levels of hCLP46 and Smad3 were examined by real-time RT-PCR in control or 0.5 μg/ml Tet treated 293TRex-hCLP46 cells for 24h. (B) 293TRex Cells were transfected with control or hCLP46 specific siRNA for 72h and then mRNA levels of hCLP46 and Smad3 were determined. n=4 in each group. * indicates p<.05 comparing control and hCLP46 overexpressed or hCLP46 siRNA transfected cells.

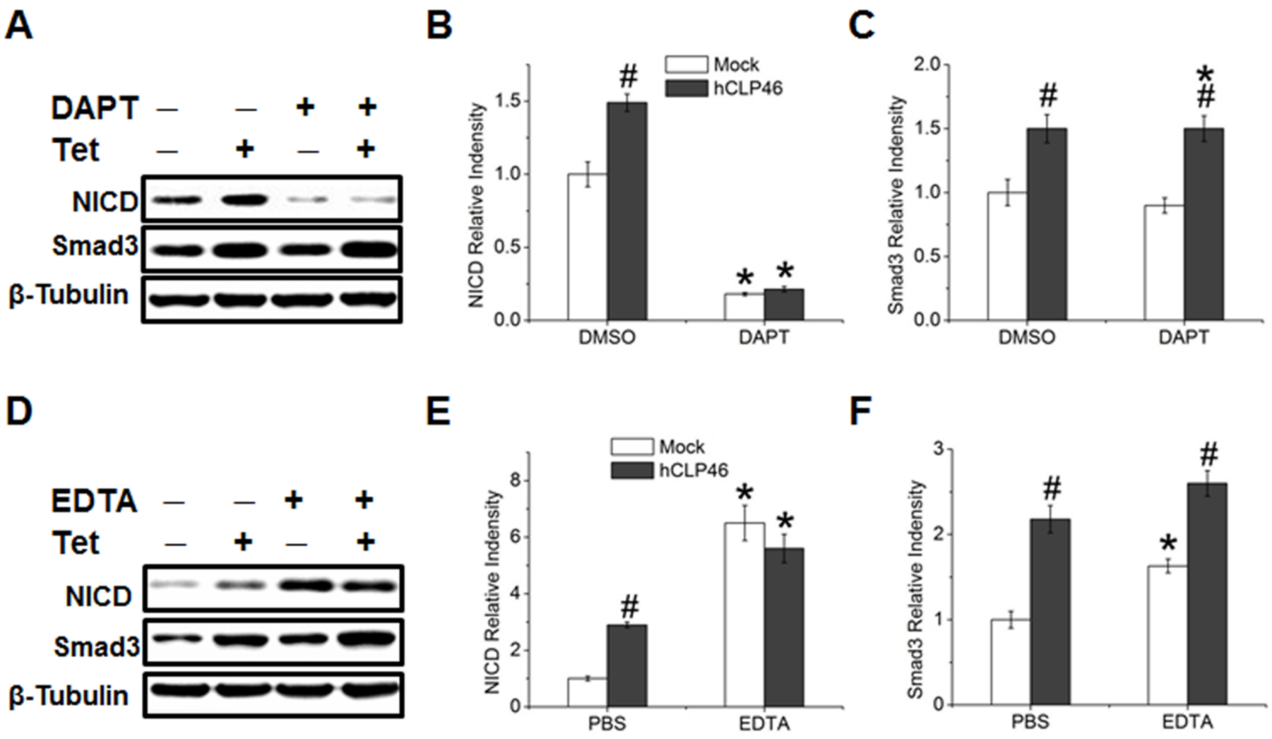

Figure S2. The up-regulation of Smad3 induced by hCLP46 overexpression is not affected by Notch signaling. 293TRex-hCLP46 cells were cultured in absence or presence of 0.5 μg/ml Tet for

24h and 2 μM DAPT or 0.5% DMSO were then added for 12h. Total cell lysates were probed for NICD and Smad3. β-Tubulin was used as a loading control (A-C). Or cells were treated with 5 mM EDTA or PBS for 30min, then replaced with fresh DMEM plus 10% FBS and cultured for additional 6h. Cell lysates were examined by western blot for NICD and Smad3 (D-F). Figures are chosen as the representative of three independent experiments. * indicates $p<.05$ comparing control and DAPT or EDTA treated cells. # indicates $p<.05$ comparing control and hCLP46 overexpressed cells.

References

Chu, Q., Liu, L., and Wang, W. (2013). Overexpression of hCLP46 enhances Notch activation and regulates cell proliferation in a cell type-dependent manner. Cell Prolif 46, 254-262.

Ma, W., Du, J., Chu, Q., Wang, Y., Liu, L., Song, M., and Wang, W. (2011). hCLP46 regulates U937 cell proliferation via Notch signaling pathway. Biochem Biophys Res Commun 408, 84-88.
